# The Role of Mothers’ Psychiatric Symptoms, Practices of Emotion Socialization and Emotion Regulation among Children Diagnosed with Attention Deficit and Hyperactivity Disorder

**DOI:** 10.1007/s10578-025-01808-4

**Published:** 2025-03-22

**Authors:** Feray Tarımtay Altun, Medine Yazıcı, Özalp Ekinci

**Affiliations:** 1 Department of Clinical Psychology, University of Health Sciences, Istanbul, Turkey; 2 Department of Psychiatry, University of Health Sciences, Erenkoy Mental Health and Neurological Diseases Education and Research Hospital, Istanbul, Turkey; 3 School of Medicine, University of Health Sciences, Istanbul, Turkey

**Keywords:** Attention deficit and hyperactivity disorder, Borderline personality disorder, Emotion regulation, Emotion socialization

## Abstract

This study investigates the mediating role of mothers’ emotion socialization practices and emotion regulation difficulties in the relationship between maternal attention deficit and hyperactivity disorder (ADHD) and borderline personality disorder (BPD) symptom levels and their children’s ADHD symptom levels. The study included 90 children (*M* = 9.71 years, *SD* = 1.33) diagnosed with ADHD and their mothers. Mothers completed Adult Attention Deficit and Hyperactivity Disorder Self-Report Scale, Borderline Personality Inventory, DSM-IV Based Disruptive Behavior Disorders Rating Scale, Coping with Children’s Negative Emotions Scale, Difficulties in Emotion Regulation Scale-Brief Form. Children filled out Cognitive Emotion Regulation Questionnaire for Children. The findings revealed that mothers’ punitive and distress reactions mediated the relationship between mothers’ and their children’s ADHD symptom levels. Furthermore, all unsupportive emotion socialization strategies mediated the relationship between mothers’ BPD symptoms and their children’s ADHD symptoms. These results indicate that unsupportive maternal responses may contribute to the severity of children’s ADHD symptoms.

## Introduction

Attention Deficit Hyperactivity Disorder (ADHD) is one of the most prevalent childhood conditions and a leading cause for referrals to child psychiatry clinics globally [1] (Elberling et al., 2015). Epidemiological studies report a male-to-female ratio of approximately 2:1 for ADHD prevalence [2]. However, in clinical samples, this ratio tends to increase, ranging from 3:1 to 5:1, and sometimes even reaching 9:1 in certain studies [3]. This discrepancy is often attributed to boys exhibiting more externalizing problems (e.g., behavioral issues), which are more noticeable and thus more likely to lead to clinical referrals. In contrast, girls often present with internalizing problems (e.g., inattention, depression, anxiety), which may be less apparent [4].

Childhood ADHD significantly increases the risk of academic, behavioral, and social difficulties [5]. Since ADHD often persists into adulthood, affecting multiple life areas such as work, relationships, and parenting [6], understanding its associated factors is crucial. Genetic factors account for about 76% of ADHD etiology [7], yet environmental influences, including parental characteristics, significantly affect the disorder’s severity and prognosis [8, 9]. Exploring the interplay between parental and child psychopathology is critical, particularly for neurodevelopmental disorders with high genetic transmission, like ADHD, as it highlights modifiable environmental factors that can enhance treatment outcomes.

### Maternal Psychopathology, Child ADHD, and Emotion Regulation

Emotion regulation involves the process of experiencing, managing, and modifying emotions as well as their behavioral expression [10, 11]. Effective emotion regulation helps initiate adaptive behaviors, engage in goal-directed activities, reduce stress levels, and prevent maladaptive responses [12]. Gratz and Roemer [13] conceptualize emotion regulation as a multidimensional process, emphasizing emotional awareness, understanding, acceptance, impulse control, and the ability to use various strategies flexibly to modulate emotions. Emotion regulation difficulties, on the other hand, refer to the dysfunctional experience, interpretation, and maladaptive responses to emotional states, which impair functioning [14].

Research suggests that parents of children with ADHD are more likely to exhibit higher rates of psychiatric conditions, including ADHD, anxiety, depression, substance abuse, and personality disorders [15]. Among these disorders, ADHD and borderline personality disorder (BPD) frequently co-occur in adults, with both disorders sharing core characteristics such as impulsivity and emotion regulation difficulties [16]. Deficits in emotion regulation have significant consequences for daily functioning [17, 18]. Parents with traits of BPD may struggle to respond empathetically to their children, maintain a stable and secure home environment, and utilize effective parenting strategies. These struggles can lead to feelings of inadequacy in their parenting role, which further undermines their ability to parent effectively [19], leading to negative outcomes within the family [20–23]). Similarly, ADHD symptoms in parents can disrupt cognitive processes, self-regulation, and motivation, thereby diminishing both parental competence and satisfaction [22, 24] (Sonuga-Barke et al., 2002). This can, in turn, exacerbate negative outcomes for both the parent and child [23, 25]. Research has established links between both parental ADHD and BPD traits and the severity of ADHD symptoms in children [20, 26–29].

Children diagnosed with ADHD often face challenges in emotion regulation [30] (Braaten & Rosen, 2000; Bunford et al., [17, 31, 32]. For instance, in a study of 8,681 children and adolescents with an ADHD diagnosis, approximately one-third were found to have difficulties regulating their emotions [33]. Studies suggest that one of the mechanisms linking parental psychopathology to children’s symptoms is the transmission of emotion regulation difficulties within the family [34, 35]. This highlights the importance of addressing emotion regulation within family-based interventions, especially when parental mental health issues are present. Parents who struggle with emotion regulation often respond to their children’s negative behaviors with overly controlling approaches or emotional disengagement. These types of parenting behaviors are linked to the development of maladaptive emotion regulation strategies in children [36]. Emotional dysregulation in parents often hinders their ability to respond effectively to their children’s emotional needs, leading to negative impacts on the child’s emotional development [37, 38]. In addition, emotional challenges of parents with ADHD or BPD can have significant implications for their children’s emotional and behavioral development, as these maladaptive strategies may be passed on or modelled by their children.

### Emotion Socialization in Maternal and Child Psychopathology

Parental attitudes toward children’s emotional expressions play a key role in children’s emotional development [39, 40]. Eisenberg et al. [41] emphasize that parental emotion socialization approaches—how parents respond to their children’s emotions—significantly influence the development of children’s emotional and social competence. Parents engage in emotion socialization through three main pathways: (a) their responses to children’s emotions, (b) discussions about emotions, and (c) parental emotional expression [41, 42].

Supportive parental responses, such as validating and encouraging emotional expression, promote better emotion regulation, emotional awareness, and social competence in children [43]. In contrast, unsupportive responses—such as punitive or dismissive reactions to negative emotions like anger or sadness—signal to the child that these emotions should be suppressed. This can lead to increased emotional arousal, prolonged negative emotional states, and difficulties in regulating behaviors [44]. Such patterns are linked to poorer emotional regulation and behavioral problems, ultimately increasing the risk for psychopathology [45, 46]. Research suggests that there is a significant relationship between psychopathology symptoms in parents and unsupportive emotion socialization practices [35, 47–49].

In addition, parents of children with clinical diagnoses, such as ADHD, tend to exhibit more unsupportive emotional socialization behaviors compared to parents of typically developing children [50, 51]. Children with ADHD often face greater emotional challenges than their typically developing peers, struggling with problem-solving when upset and focusing more on negative aspects of situations [52]. As a result, unsupportive parental behaviors can exacerbate these difficulties or increase the risk of related issues [53]. A few studies investigating emotion socialization practices of mothers’ of children with ADHD suggest that supportive emotion socialization approaches are linked to improvements in both emotional regulation and social functioning in these children [54–57]. An emotion socialization program developed for families of hyperactive preschool-aged children includes components such as teaching parents emotion socialization strategies to enhance children’s emotion regulation such as helping children identify and label emotions, managing negative emotions, promoting positive emotional experiences, and modeling emotion regulation and expression for children [56]. The results of this study largely confirmed the program’s effectiveness in decreasing ADHD symptom levels, reducing children’s emotional instability, and enhancing parental emotion socialization practices. Another study conducted with young children and their mothers evaluated the effectiveness of an emotion socialization program and reported that enhancing mothers’ emotion regulation skills and supportive emotion socialization approaches was associated with a reduction in behavioral problems in children [58].

### The Current Study

Although previous studies have explored the relationship between mothers’ psychiatric symptoms and their emotion socialization approaches, the specific link between ADHD and BPD symptoms in mothers of children diagnosed with ADHD and these socialization strategies remains largely unexamined. Moreover, research on the cognitive emotion regulation strategies employed by school-aged children diagnosed with ADHD is limited. Investigating this area will help fill a critical gap in the literature, providing insights into both the cognitive strategies employed by children and the parenting dynamics influenced by ADHD and BPD symptoms in their parents. The hypotheses are as follows:

#### H1

Mothers’ emotion socialization approaches mediate the relationship between mothers’ ADHD and BPD symptom levels and children’s ADHD symptom levels.

#### H2

Mothers’ emotion regulation difficulties mediate the relationship between mothers’ ADHD and BPD symptom levels and children’s ADHD symptom levels.

#### H3

Children’s emotion regulation strategies mediate the relationship between mothers’ emotion socialization approaches and children’s ADHD symptom levels.

## Method

### Participants

Participants included 90 Turkish children (67 boys; 23 girls) aged 8–12 years (*M* = 9.71, *SD* = 1.33) diagnosed with ADHD and their mothers. When evaluating the ADHD profiles of the child participants, it was found that 27.8% (*n* = 25) had a predominantly inattentive type, while 12.2% (*n* = 11) exhibited a predominantly hyperactive-impulsive type, and 60% (*n* = 54) had a combined type. Additionally, 93.3% (*n* = 84) of the participants were taking medication, with 6.7% (*n* = 6) not using any medication. Regarding the types of medication used for ADHD, 90% (*n* = 81) were using methylphenidate, 3.3% (*n* = 3) were using atomoxetine, and 6.7% (*n* = 6) were not on any medication. In terms of comorbid diagnoses, 13.3% (*n* = 12) of the children had an anxiety disorder, 3.3% (*n* = 3) had conduct disorder, 8.9% (*n* = 8) had a specific learning disability, and 73.4% (*n* = 66) did not have any comorbid diagnoses. Mothers’ age ranged from 28 to 53 (M = 38.6, SD = 5.6). The education levels of the mothers were also assessed: 22.2% (*n* = 20) had completed primary education, 16.7% (*n* = 15) secondary education, 33.3% (*n* = 30) high school, 5.6% (*n* = 5) had an associate degree, 21.1% (*n* = 19) held a bachelor’s degree, and 1.1% (*n* = 1) had a graduate-level education. When the employment status of the mothers is examined, it was found that 68.9% (*n* = 62) are not working and 31.1% (*n* = 28) are working.

### Procedure

The study included children who were diagnosed with ADHD according to DSM-5 diagnostic criteria and their mothers. The participants in this study were recruited from the Child and Adolescent Psychiatry Clinic of the University of Health Sciences Erenkoy Research Hospital, located in Istanbul, Turkey. The hospital is situated in a densely populated urban area and serves a diverse population including individuals from both urban and rural areas. The inclusion criteria for children were 8–12 years of age and having no DSM-5 diagnosis of autism spectrum disorder, bipolar disorder, neurological disorders, and intellectual disability. For mothers, inclusion criteria were having no known diagnosis of bipolar disorder, psychotic disorders, intellectual disability, and dementia. Eligible children and their mothers were referred to the researcher after undergoing psychiatric evaluations by a child and adolescent psychiatrist. Information about the study was provided and written informed consent was obtained from mothers who voluntarily agreed to participate and assent was obtained from all children included in the study. Mothers completed Adult Attention Deficit and Hyperactivity Disorder Self-Report Scale, Borderline Personality Inventory, DSM-IV Based Disruptive Behavior Disorders Rating Scale, Coping with Children’s Negative Emotions Scale, Difficulties in Emotion Regulation Scale-Brief Form. Children filled out the Cognitive Emotion Regulation Questionnaire for Children.

### Instruments

#### Adult Attention Deficit and Hyperactivity Disorder Self-Report Scale

The World Health Organization [59] developed the Adult ADHD Self-Report Scale (ASRS) to screen for ADHD symptoms in adults. It consists of 18 items rated on a 4-point Likert scale (1: *Never*, 4:) and includes two subscales: inattention and hyperactivity/impulsivity. The Turkish adaptation was conducted by Doğan et al. [60], with a Cronbach’s alpha of 0.88, indicating good reliability. In this study, it was used to assess mothers’ ADHD symptoms and Cronbach’s alpha was 0.91 in this study.

#### The Borderline Personality Inventory (BPI)

The scale was developed by Leichsenring [61], is a self-report tool designed to measure borderline personality traits. It includes 53 items, answered as true/false, and the total score is calculated from the first 51 items. The scale covers four subscales: identity diffusion, primitive defenses, impaired reality testing, and fear of closeness. The Cronbach’s alpha values range from 0.68 to 0.91. The Turkish validity and reliability study was conducted by Aydemir et al. [62], and the Cronbach’s alpha was reported as 0.92. In this study, it was used to assess mothers’ BPD symptoms and Cronbach’s alpha was 0.90 in this study.

#### DSM-IV Disruptive Behavior Disorders Rating Scale

DSM-IV Disruptive Behavior Disorders Rating Scale was developed by Turgay [63] and translated by Ercan et al., [64] into Turkish. The scale consists of 41 items in total, including 9 items for inattention, 9 items for hyperactivity and impulsivity, 8 items for oppositional defiant disorder (ODD), and 15 items for conduct disorder (CD). It is scored using a 4-point Likert scale (0: *Never*, 3: *Very Much*). In this study, only the subscales for inattention and hyperactivity-impulsivity and total ADHD score were used to assess the level of ADHD symptoms in children. Cronbach’s alpha was 0.93 for total ADHD scale in this study.

#### The Coping with Children’s Negative Emotions Scale (CCNES)

It was developed by Fabes and colleagues [65] to assess parents’ emotion socialization approaches. It includes 12 scenarios that depict children’s negative emotions such as anger, fear, shame, sadness, and frustration. For each scenario, there are six items evaluating different parental reactions. The scale contains six subscales: emotion-focused, problem-focused, expressive encouragement, punitive, minimizing, and distress-reaction responses. Responses are rated on a 5-point Likert scale (1: *Never*, 5: *Always*). The original scale’s Cronbach’s alpha values range from 0.69 to 0.85. The Turkish adaptation by [66] Yağmurlu et al. (2010) reports Cronbach’s alpha values between 0.54 and 0.88. In this study, Cronbach’s alpha levels of the subscales ranged from 0.54 to 0.88 in this study.

#### The Difficulties in Emotion Regulation Scale-Short Form (DERS-16)

The scale was developed by Bjureberg et al. [67] and assesses five dimensions of emotion regulation difficulties: clarity, goals, impulse, strategies, and non-acceptance. It consists of 16 items rated on a 5-point Likert scale (1: *Almost Never*, 5: *Almost Always*), with a reported Cronbach’s alpha of 0.92. Higher scores indicate greater difficulties in emotion regulation. In this study, the scale was filled out by mothers and used to evaluate mothers’ difficulties in emotion regulation. The Turkish adaptation, by Yiğit and Güzey Yiğit [68], also found a Cronbach’s alpha of 0.92. Cronbach’s alpha of the scale was 0.95 in this study.

#### The Cognitive Emotion Regulation Questionnaire for Children (CERQ-C)

The scale was developed by Garnefski and Kraaij [69] to assess children’s cognitive emotion regulation strategies and filled out by children. It contains 36 items divided into nine subscales, including self-blame, acceptance, rumination, positive refocusing, planning, positive reappraisal, putting into perspective, catastrophizing, and other-blame. Responses are rated on a 5-point Likert scale (1: *Never*, 5: *Always*), with Cronbach’s alpha values between 0.62 and 0.79. The Turkish adaptation by [70] Akfırat and Turan (2021) found Cronbach’s alpha values between 0.43 and 0.80. In this study, Cronbach’s alpha values were between 0.49 and 0.78.

### Data Analysis

All analyses were carried out using IBM SPSS Statistics software, version 24. Pearson’s Correlation was used for correlation analyses. For comparisons of the scale scores across ADHD subtypes, one-way ANOVA (Analysis of Variance) was conducted. Post-hoc analyses were applied in cases where significant differences were observed, considering the homogeneity of variances. In the present study, The Variance Inflation Factor (VIF) values for the predictor variables in the mediation models were all below the threshold of 10, and the tolerance values were above 0.10, indicating that there was no significant multicollinearity among the variables [71]. All the mediational relationships were estimated by the Hayes’ Process macro using bootstrap analysis through 5000 samplings. Mediation effects were determined based on the 95% confidence intervals, with the intervals not containing zero [72].

## Results

### Preliminary Analyses

Descriptive statistics and correlations of the study variables are presented in Table 1. The mean score on the Borderline Personality Inventory (M = 8.96) in the current study was below the clinical cut-off score of 15/16, suggesting that, on average, the participants did not exhibit clinically significant levels of borderline personality traits. For the remaining measures, no established cut-off scores exist; therefore, the results are interpreted based on relative scores (e.g., mean, SD, range).

For group comparisons (predominantly inattentive type, predominantly hyperactive-impulsive type, and combined ADHD types of children) of mothers’ emotion socialization strategies, one-way ANOVA tests showed significant differences in mothers’ dismissive responses across groups (*F*(2, 87) = 4.885, *p* =.010). LSD (The Least Significant Difference) post hoc tests indicated that mothers of children with predominantly inattentive type exhibited significantly lower dismissive responses than those of children with combined type (Mean_diff_ = − 0.55, 95% CI [-0.92, − 0.17], *p* =.005). Regarding children’s cognitive emotion regulation strategies, self-blame scores differed significantly across groups (*F*(2, 87) = 3.586, *p* =.032). According to the LSD post hoc test results, children in the predominantly inattentive group had significantly higher self-blame scores compared to those in the predominantly hyperactive-impulsive group (Mean_diff_ = 3.14, 95% CI [0.75, 5.54], *p* =.011). In addition, it was found that the self-blame scores of children in the combined group were also significantly higher than those in the predominantly hyperactive-impulsive group (Mean_diff_ = 2.62, 95% CI [0.43, 4.81], *p* =.020). In addition, acceptance scores differed significantly across groups (*F*(2, 87) = 3.546, *p* =.033). According to the LSD post hoc test, participants in the predominantly inattentive group had significantly higher acceptance scores compared to those in the predominantly hyperactive-impulsive group (Mean_diff_ = 3.26, 95% CI [0.75, 5.77], *p* =.011). Similarly, children in the combined group also showed significantly higher acceptance scores than those in the predominantly hyperactive-impulsive group (Mean_diff_ = 2.75, 95% CI [0.45, 5.04], *p* =.019).

### Mediation Analyses

Reported direct effects include standardized regression coefficients and unstandardized confidence intervals; indirect effects are accompanied by standardized regression coefficients and standardized confidence intervals.

**Table 1:** Descriptive statistics and intercorrelations of the study variables

Variable	1	2	3	4	5a	5b	5c	5d	5e	5f	6a	6b	6c	6d	6e	6f	6 g	6 h	6ı
1. T-DSM-IV-S	1																		
2. ASRS	0.394**	1																	
3. BPI	0.270**	0.460**	1																
4. DERS-16	0.357**	0.447**	0.537**	1															
CCNES																			
5a. distress	0.468**	0.387**	0.252*	0.474**	1														
5b. punitive	0.394**	0.230*	0.386**	0.405**	0.576**	1													
5c.minimizing	0.364**	0.033	0.221*	0.200	0.290**	0.697**	1												
5d. expressive encouragement	0.092	0.119	− 0.047	0.002	0.041	0.049	0.158	1											
5e. emotion focused	0.194	0.100	0.006	− 0.001	− 0.034	0.056	0.348**	0.719**	1										
5 f. problem focused	0.300**	0.171	0.067	0.090	0.106	0.090	0.388**	0.686**	0.750**	1									
CERQ-k																			
6a. self-blame	0.134	0.168	0.259*	0.229*	0.033	0.218*	0.169	0.017	0.118	0.102	1								
6b. acceptance	0.125	0.075	0.177	− 0.012	− 0.151	0.163	0.195	0.150	0.334**	0.187	0.639**	1							
6c.rumination	0.058	0.241*	0.286**	0.321**	0.146	0.089	0.082	0.338*	0.152	0.299**	0.341**	0.173	1						
6d. positive refocussing	0.120	− 0.148	0.090	0.009	0.190	0.181	0.292**	0.177	0.107	0.183	− 0.005	− 0.065	0.300**	1					
6e. planning	− 0.100	− 0.009	− 0.049	− 0.071	0.045	0.097	160	0.345**	0.165	0.207*	0.094	0.015	0.381**	0.452**	1				
6 f. positive reappraisal	0.182	000	− 0.070	0.084	0.057	0.025	0.092	0.419**	0.351**	0.275**	0.067	0.067	0.375**	0.279**	0.425**	1			
6 g. putting into perspective	0.123	0.110	0.083	0.088	0.055	0.140	0.091	0.130	0.103	0.169	0.299**	0.177	0.153	0.249*	0.488**	0.329**	1		
6 h. catastrophising	0.092	0.131	0.157	0.102	− 0.148	0.075	0.027	0.074	0.081	0.181	0.637**	0.504**	0.350**	-,011	,143	,121	0.438**	1	
6ı. other-blame	0.275**	0.173	0.167	0.236*	0.128	0.271**	0.245*	0.099	0.142	0.290**	0.251*	0.280**	0.231*	0.196	0.060	0.160	0.424**	0.473**	1
*M (SD)*	32.61	24.54	8.96	32.3	2.46	2.06	2.87	3.98	4.17	4.15	9.9	11.01	13.17	14.04	14.17	12.08	11.57	11.09	11.11
	12.86	12.86	7.57	14.7	0.60	0.74	0.82	0.67	0.67	0.57	3.43	3.58	4.03	3.54	3.66	3.19	3.84	4.06	4.03
*Range*	7–54	0–55	0–37	12–75	1.11–4.11	1-4.50	1.50–4.75	2.17-5	2–5	2.17-5	4–19	4–20	4–20	4–20	5–20	4–20	4–20	4–20	4–20

T-DSM-IV-S: DSM-IV Disruptive Behavior Disorders Rating Scale ADHD Total Score, ASRS: Adult Attention Deficit and Hyperactivity Disorder Self-Report Scale Total Score, BPE: Borderline Personality Inventory Total Score, Difficulties in Emotion Regulation Short Form Total Score, CCNES: Coping with Children’s Negative Emotions, CERQ-k: Cognitive Emotion Regulation Scale for Children

*p <.05, **p <.01

First mediation model examined the mediating role of mothers’ punitive reactions in the relationship between mothers’ ADHD symptom levels and children’s ADHD symptom levels. The model was significant (*F*(2, 87) = 14.702, *p* =.000, *R²* = 0.252), explaining approximately 25% of the variance in children’s ADHD scores. Results showed a significant total indirect effect (*β* = 0.074, SE = 0.037, 95% CI [0.006, 0.152]) and revealed that punitive reactions were found to significantly mediate the relationship.

Second mediation model examined the mediating role of mothers’ distress reactions in the relationship between mothers’ ADHD symptom levels and children’s ADHD symptom levels. This model was also significant (*F*(2, 87) = 16.252, *p* =.000, *R²* = 0.272), explaining 27% of the variance in children’s ADHD scores. In addition, mothers’ distress reactions significantly mediated the relationship (*β* = 0.143, SE = 0.047, 95% CI [0.061, 0.242]).

Third mediation model investigated the mediating role of mothers’ punitive reactions between mothers’ BPD symptom levels and children’s ADHD symptom levels and the model was significant, (*F*(2, 87) = 9.036, *p* =.000, *R²* = 0.172) with punitive reactions acting as a significant mediator (*β* = 0.131, SE = 0.045, 95% CI [0.044, 0.230]).

Forth mediation model examined mothers’ distress reactions as a mediator in the relationship between mothers’ BPD symptom levels and children’s ADHD symptom levels. The model was significant (*F*(2, 87) = 13.993, *p* =.000, *R²* = 0.243), explaining 24% of the variance in children’s ADHD symptom levels, and distress reactions were a significant mediator (*β* = 0.108, SE = 0.038, 95% CI [0.037, 0.189]).

Fifth model examined dismissive reactions as a mediator in the relationship between mothers’ BPD symptom levels and children’s ADHD symptom levels. This model was significant (*F*(2, 87) = 8.941, *p* =.000, *R²* = 0.17), with dismissive reactions mediating the relationship (*β* = 0.108, SE = 0.038, 95% CI [0.037, 0.189]).

Model 6 assessed mothers’ emotion regulation difficulties as a mediator between mothers’ ADHD symptom levels and children’s ADHD symptom levels. The model was significant (*F*(2, 87) = 10.626, *p* =.000, *R²* = 0.196), However, total indirect effect of mothers’ emotion regulation difficulties was not significant (*β* = 0.101, SE = 0.056, 95% CI [-0.014, 0.208]).

Model 7 examined mothers’ emotion regulation difficulties between mothers’ BPD symptom levels and children’s ADHD symptoms levels. The model was significant (*F*(2, 87) = 6.874, *p* =.002, *R²* = 0.136), explaining %14 of variance in children’s ADHD symptoms. There was a significant indirect effect (*β* = 0.160, SE = 0.066, 95% CI [0.030, 0.289]), meaning a significant mediator effect of mothers’ emotion regulation difficulties in the relationship between mothers’ BPD scores and children’s ADHD scores.

Model 8 investigated children’s other-blame cognitive regulation strategy as a mediator between mothers’ punitive reactions and children’s ADHD symptom levels. Although the model was significant (*F*(2, 87) = 9.950, *p* =.000, *R²* = 0.186), the mediation effect was not significant (*β* = 0.049, SE = 0.034, 95% CI [-0.003, 0.130]).

**Fig. 1:**
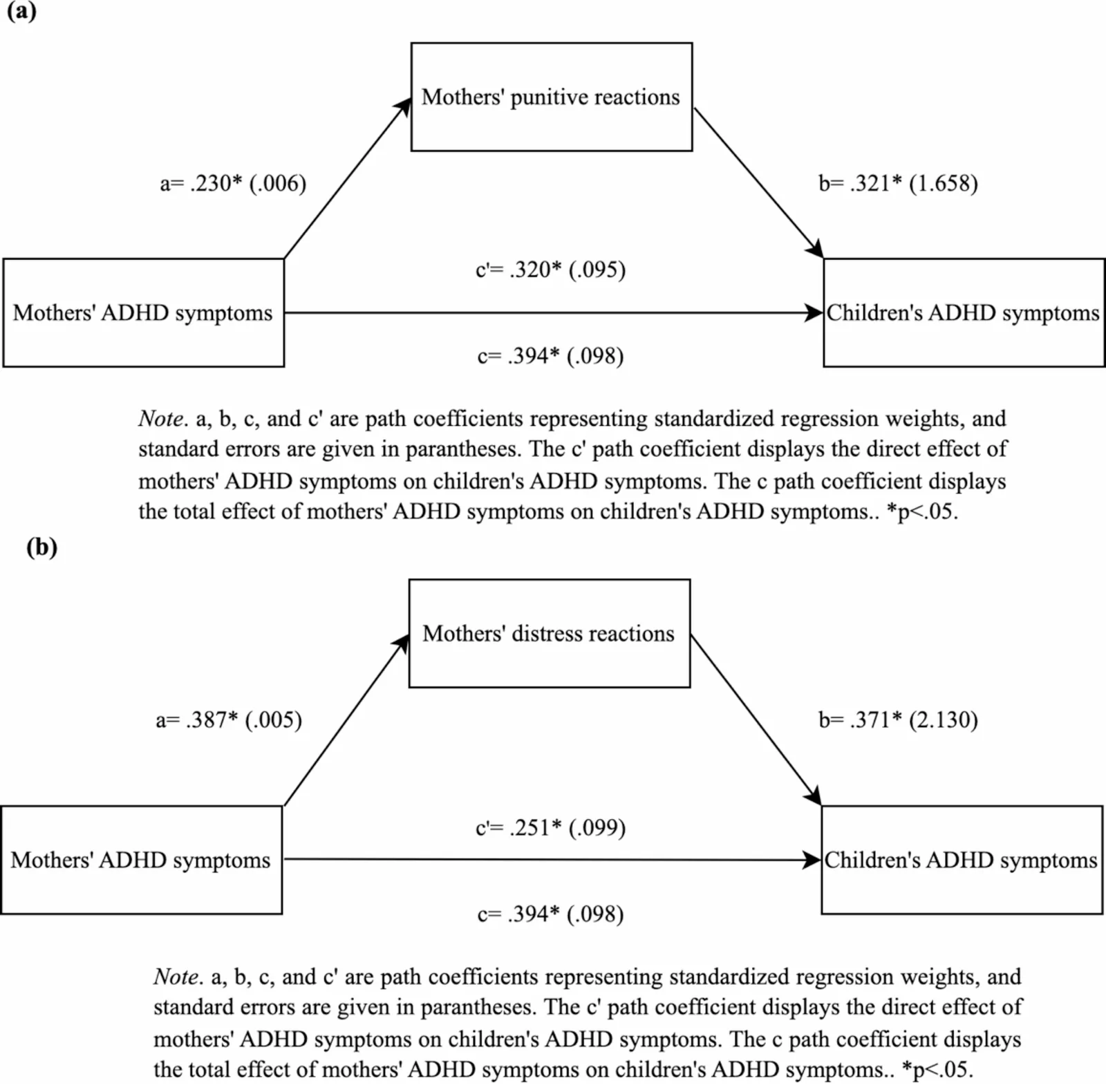
**a**: Model 1 **b**: Model 2

**Fig. 2:**
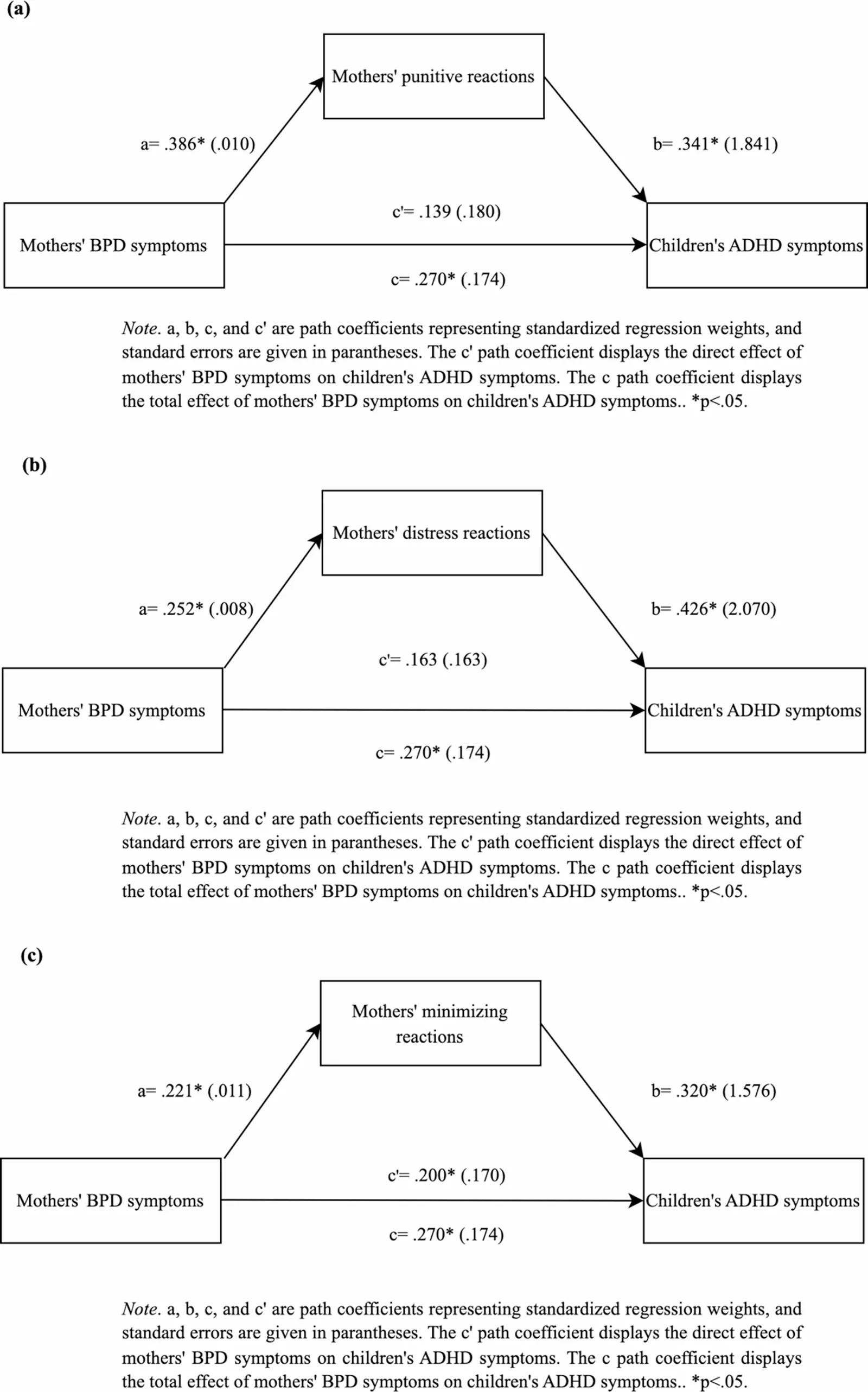
**a**: Model 3 **b**: Model 4 **c**: Model 5

**Fig. 3:**
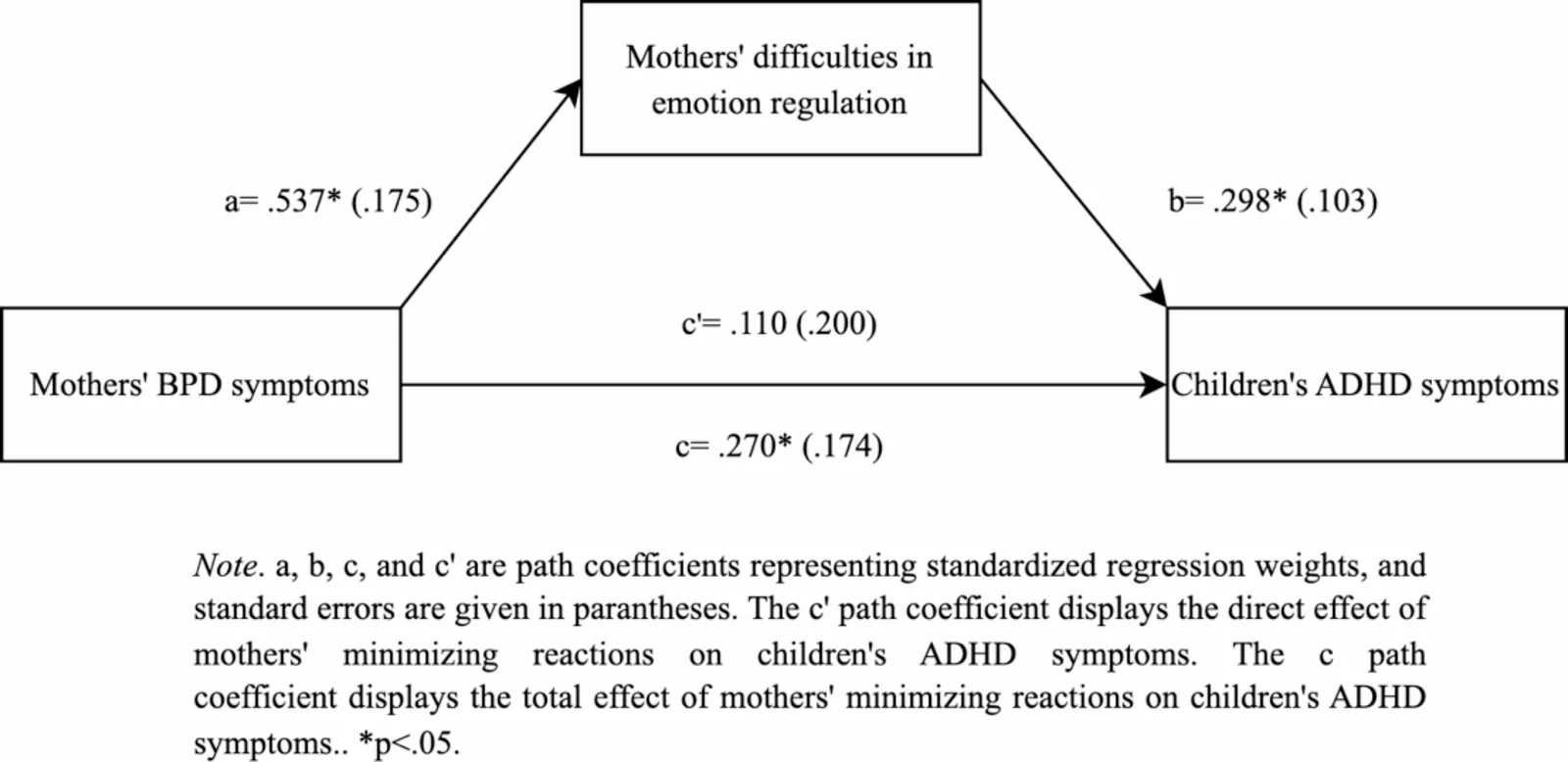
Model 7

**Fig. 4:**
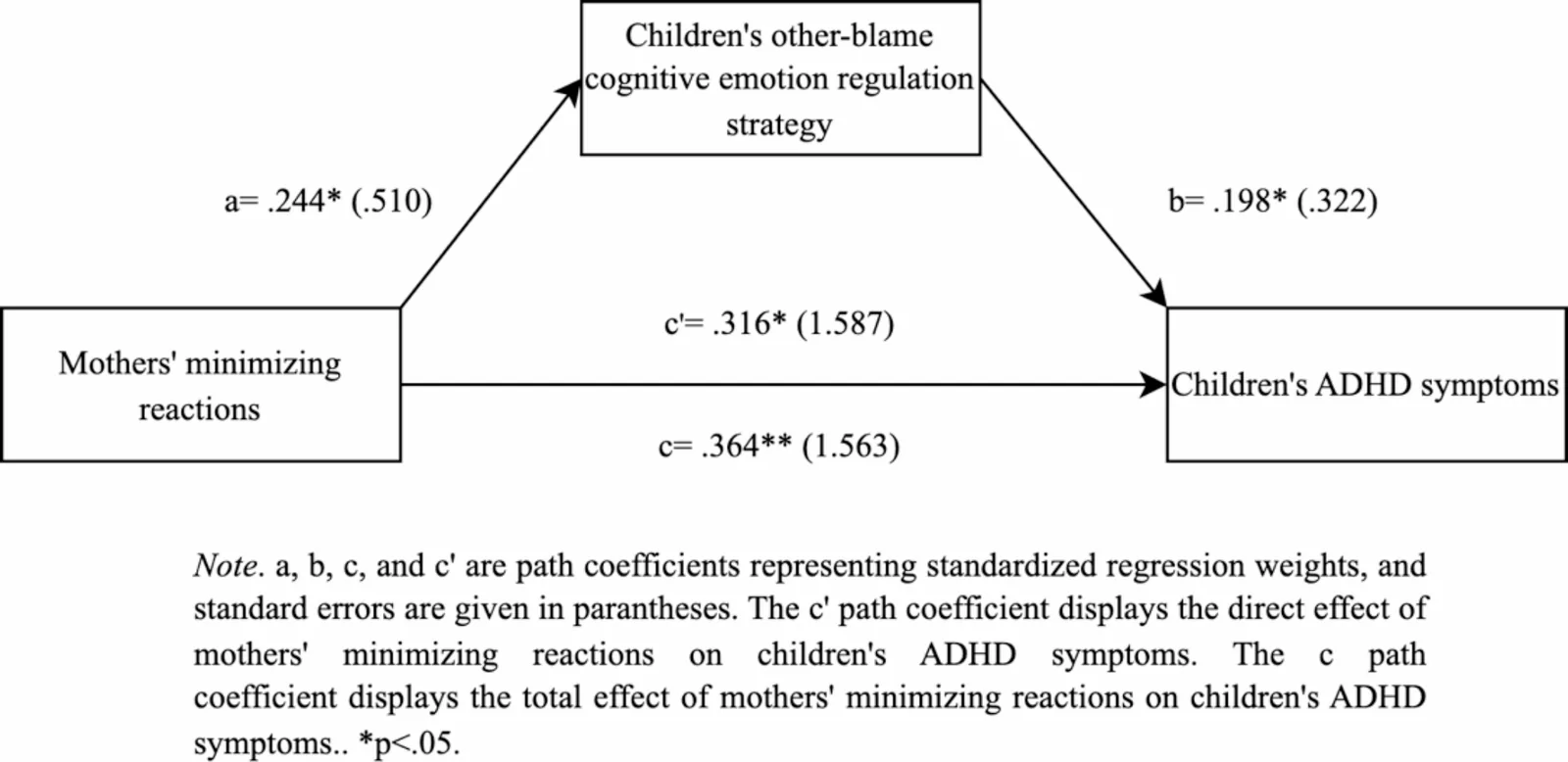
Model 9

Model 9 tested the role of children’s other-blame cognitive regulation strategy in the relationship between mothers’ dismissive reactions and children’s ADHD symptom levels. The model was significant (*F*(2, 87) = 9.869, *p* =.000, *R²* = 0.169). Also, total indirect effect was significant the mediation was significant (*β* = 0.048, SE = 0.036, 95% CI [0.000, 0.136]), revealing that children’s other-blame strategy mediates the relationship between mothers’ dismissive reactions and children’s ADHD symptom levels. Mediated models can be seen in Figs. 1, 2 and 3, and 4.

## Discussion

The study aimed to examine the mediating role of mothers’ emotion regulation difficulties and emotion socialization approaches in the relationship between the levels of ADHD and BPD symptoms in mothers of children diagnosed with ADHD and their children’s ADHD symptom levels. Additionally, cognitive emotion regulation strategies of the children were assessed, and the mediating role of children’s cognitive emotion regulation strategies in the relationship between mothers’ emotion socialization approaches and children’s ADHD symptom levels was tested.

It was first hypothesized that mothers’ emotion socialization approaches would mediate the relationship between mothers’ ADHD and BPD symptom levels and children’s ADHD symptom levels. The results indicate that mothers’ punitive and distress-worry reactions mediate the relationship between mothers’ ADHD symptoms levels and their children’s ADHD symptom levels. These findings align with previous research that highlights the mediating role of negative parenting behaviors in the relationship between parental ADHD symptoms and child ADHD symptoms Breaux, Brown, & Harvey [47], Tung et al., [73]. ADHD symptoms, particularly inattention and impulsivity, may impair mothers’ parenting abilities, making it difficult for them to manage emotions and respond appropriately, especially when interacting with children with ADHD. This, in turn, may exacerbate the severity of ADHD symptoms in children.

Furthermore, the study found that mothers’ dismissive, punitive, and distress-worry reactions mediated the relationship between mothers’ BPD symptom levels and their children’s ADHD symptom levels. Consistent with previous research, mothers with elevated BPD symptoms were found to use more negative emotion socialization strategies, such as dismissive and punitive responses [74]. These parents may struggle with emotional regulation, recognition of others’ emotions, and impulse control, which could lead to maladaptive responses in interactions with their ADHD-diagnosed children. The use of such strategies might impair children’s ability to manage negative emotions, potentially increasing behavioral problems [38]; [41]; Bjork et al., 2020; Morris et al., [75].

In addition, the study investigated the mediating role of mothers’ emotion regulation difficulties in the relationship between their ADHD and BPD symptom levels and their children’s ADHD symptom levels. The results showed that while emotion regulation difficulties did not mediate the relationship between maternal ADHD symptom levels and children’s ADHD symptom levels, emotion regulation difficulties mediated the relationship between maternal BPD symptom levels and children’s ADHD symptom levels. Parents with ADHD symptoms often face difficulties in maintaining attention and staying calm during interactions with their children, which could heighten their children’s ADHD symptoms [23]. However, the lack of significant mediation by emotion regulation difficulties in the ADHD group suggests that genetic transmission may play a stronger role rather than emotion regulation difficulties.

Conversely, the significant mediation effect found in mothers with BPD symptoms indicates that these mothers may struggle more with emotional regulation, contributing to increased ADHD symptoms in their children. Individuals with BPD tend to have difficulties both regulating their own emotions and recognizing others’ emotions [76]. Studies show that BPD individuals may misinterpret facial expressions and neutral emotional cues as negative, intensifying emotional reactions [77–79]. This may lead to experiencing stress or giving punitive reactions towards children, which could impair children’s emotion regulation and behavioral control, exacerbating ADHD symptoms. Therefore, maternal emotion regulation difficulties may play a key role in increasing children’s emotional and behavioral problems.

The study also tested the mediating role of children’s cognitive emotion regulation strategies in the relationship between maternal emotion socialization approaches and children’s ADHD symptom severity. First, the mediating role of children’s other-blame strategy between mothers’ punitive reactions and children’s ADHD symptoms was examined, but the mediation was not significant. However, other-blame strategy significantly mediated the relationship between mothers’ dismissive reactions and children’s ADHD symptom levels. This suggests that punitive and dismissive maternal responses may influence children’s emotion regulation and ADHD symptoms through different mechanisms.

Punitive emotional socialization includes parental responses such as scolding, withholding privileges (e.g., restricting outdoor play or TV time), reflecting an authoritarian parenting style [80]. These responses may suppress children’s emotions and behaviors, as seen in Eisenberg et al.‘s [41] study, where punitive parenting caused fear, leading children to suppress their emotions instead of processing them healthily. Dismissive emotion socialization, on the other hand, includes belittling or ignoring a child’s emotions, such as calling them overly dramatic. Research has linked dismissive parenting to avoidant coping in children [44], leading children to externalize responsibility for negative emotions, potentially worsening ADHD symptoms. Children’s use of the other-blame strategy investigated in the present study involves attributing responsibility for negative situations to others, believing that others are at fault while denying personal responsibility for the outcome [69]. Research suggests that blaming others is associated with behavioral problems [81] (McGee et al., 2001). Dismissive maternal reactions may encourage children to externalize blame for their difficulties, potentially exacerbating ADHD symptoms, while punitive approaches may suppress emotional expression, with both strategies potentially contributing to long-term negative consequences.

When comparing cognitive emotion regulation strategies across ADHD presentations, children with predominantly inattentive or combined group used self-blame cognitive emotion regulation strategy more than those with predominantly hyperactive-impulsive group. They also employed acceptance strategy more frequently. This aligns with research indicating that inattentive symptoms more strongly predict internalizing problems like depression and anxiety [82]. In addition, acceptance, while helpful in some contexts, can lead to helplessness if misapplied, as described in learned helplessness theory [83]. For ADHD children facing repeated failures, acceptance may sometimes hinder problem-solving, fostering passivity [84]. This highlights the need for clinical interventions that address their tendencies to internalize, helping them develop more functional coping strategies in response to negative events.

Based on the correlation analysis, a positive relationship was found between mothers’ ADHD symptom levels and their distress-worry and punitive responses to their children’s negative emotions. Furthermore, a positive and significant relationship was observed between mothers’ BPD symptom levels and their punitive, dismissive, and distress-worry responses. These findings suggest that in a clinical population of mothers with ADHD or BPD, these associations may be even stronger, potentially leading to more pronounced disruptions in emotion socialization strategies.

In examining mothers’ emotion socialization approaches related to children’s ADHD presentations, mothers of children with combined ADHD exhibited higher levels of dismissive strategies compared to those of children with predominantly inattentive ADHD. This can be linked to the more intense negative emotions and regulation difficulties faced by children with combined ADHD due to their hyperactive and impulsive behaviors. Maedgen and Carlson [31] also found that children with combined ADHD experienced emotions more intensely and struggled more with behavior control than their predominantly inattentive peers. Consequently, parents may respond dismissively or misinterpret their children’s emotional expressions, think that they are misbehaving or being clumsy and fail to address their emotional needs appropriately.

### Limitations

When interpreting the study’s findings, a few limitations must be considered. First, the cross-sectional design of the study limits the ability to establish causal relationships. Research suggests a reciprocal relationship between children’s externalizing behaviors and parenting, where children’s behavioral difficulties increase negative parenting, which in turn worsens the child’s problems [85–87]. Second, the study relied on self-report scales without incorporating observational tools, potentially affecting the objectivity of the responses. Future research could include observational measures to evaluate variables such as emotion socialization and emotion regulation. Third, in assessing children’s ADHD symptoms and other variables, information was gathered exclusively from mothers, under the assumption that they play a more significant role in children’s emotional and behavioral difficulties. Future studies could explore both maternal and paternal psychiatric symptoms and emotion socialization approaches, and additionally gather data from teachers when assessing children’s ADHD symptoms. Fourth, the distress reactions subscale of the CCNES used in this study demonstrated lower internal consistency reliability (α = 0.54). This limitation has been noted in previous research and the Turkish adaptation of the scale, where items with low factor loadings and face validity issues were excluded, leading to a refined subscale structure [88]. Despite these revisions, similar challenges in achieving higher internal consistency have been reported in Western samples as well [89]. The findings related to this subscale should be interpreted cautiously, taking into account its psychometric limitations and situating the results within the broader context of maternal emotional socialization strategies. Fifth, The CERQ-C demonstrated lower reliability coefficients for certain subscales such as positive reappraisal and acceptance which may have impacted the robustness of the findings related to those specific domains. Future studies should interpret results associated with these subscales with caution and consider employing alternative measures or additional reliability assessments to ensure the accuracy and consistency of the data. Lastly, while evaluating mothers’ responses to children’s negative emotions, these emotions were examined under a broad framework. Future research could examine emotion socialization responses towards specific negative emotions, such as anger, sadness, fear, frustration, and shame, in families of children with ADHD.

### Implications

The findings offer valuable insights into the impact of maternal ADHD and BPD symptoms on parenting dynamics and their children’s emotional development. First, the study reveals that unsupportive maternal emotion socialization approaches, such as punitive and dismissive reactions, mediate the relationship between maternal ADHD/BPD symptom levels and children’s ADHD symptom levels. This highlights the importance of parent training programs that aim to reduce the use of punitive and dismissive responses, promoting healthier emotion regulation strategies in children with ADHD. Second, the mediating role of maternal emotion regulation difficulties in the relationship between maternal ADHD and BPD symptoms and children’s ADHD symptoms emphasizes the need to address maternal mental health in treatment interventions, as supporting mothers’ emotion regulation can positively influence both their parenting and their children’s ADHD symptoms.

The sample in the present study predominantly consists of boys (%74.4) which likely reflects the increased visibility of ADHD symptoms in boys, which facilitates their identification and referral to clinical settings [4]. However, this also raises the concern that ADHD symptoms in girls may be underrecognized or overlooked. Therefore, it is essential to consider this gender distribution when interpreting the study findings, as it may influence the generalizability of the results. In addition, the majority of participants in the present study were receiving medication, which reflects a clinically representative sample (Hauck et al., 2017). However, this limits the generalizability of the findings to children with ADHD who are not on medication. Future research should aim to include and examine children diagnosed with ADHD who are not undergoing pharmacological treatment, to provide a more comprehensive understanding of this population. Lastly, future studies could examine mothers with clinically diagnosed ADHD or BPD to further explore how these diagnoses influence emotion socialization and child development.

## Data Availability

No datasets were generated or analysed during the current study.

## References

[1] Elberling H, Linneberg A, Rask CU, Houman T, Goodman R, Skovgaard M, A (2016) Psychiatric disorders in Danish children aged 5–7 years: a general population study of prevalence and risk factors from the Copenhagen child cohort (CCC 2000). Nord J Psychiatry 70(2):146–155. 10.3109/08039488.2015.1070199 26509656

[2] Ayano G, Demelash S, Gizachew Y, Tsegay L, Alati R (2023) The global prevalence of attention deficit hyperactivity disorder in children and adolescents: an umbrella review of meta-analyses. J Affect Disord 339:860–866. 10.1016/j.jad.2023.07.071 37495084

[3] Spetie L, Arnold EL (2018) Attention-deficit hyperactivity disorder. A Martin, MH Bloch, FR Volkmar (Eds.), Lewis’s Child and Adolescent Psychiatry: A Comprehensive Textbook

[4] Quinn PO, Madhoo M (2014) A review of attention-deficit/hyperactivity disorder in women and girls: uncovering this hidden diagnosis. Prim care Companion CNS Disorders 16(3). 10.4088/PCC.13r01596. PCC.13r01596 PMC4195638 25317366

[5] Flory K, Molina BS, Pelham WE Jr, Gnagy E, Smith B (2006) Childhood ADHD predicts risky sexual behavior in young adulthood. J Clin Child Adolesc Psychology: Official J Soc Clin Child Adolesc Psychol Am Psychol Association Div 53(4):571–577. 10.1207/s15374424jccp3504_8 17007602

[6] Katzman MA, Bilkey TS, Chokka PR, Fallu A, Klassen LJ (2017) Adult ADHD and comorbid disorders: clinical implications of a dimensional approach. BMC Psychiatry 17(1):302. 10.1186/s12888-017-1463-3 PMC5567978 28830387

[7] Faraone SV, Perlis RH, Doyle AE, Smoller JW, Goralnick JJ, Holmgren MA, Sklar P (2005) Molecular genetics of attention-deficit/hyperactivity disorder. Biol Psychiatry 57(11):1313–1323. 10.1016/j.biopsych.2004.11.024 15950004

[8] Beauchaine TP, Hinshaw SP, Pang KL (2010) Comorbidity of attention-deficit/hyperactivity disorder and early-onset conduct disorder: Biological, environmental, and developmental mechanisms. Clin Psychol Sci Pract 17(4):327

[9] Faraone SV, Larsson H (2019) Genetics of attention deficit hyperactivity disorder. Mol Psychiatry 24(4):562–575. 10.1038/s41380-018-0070-0 PMC6477889 29892054

[10] Eisenberg N, Hofer C, Vaughan J (2007) Effortful control and its socioemotional consequences. Handb Emot Regul 2:287–288

[11] Thompson RA (1994) Emotion regulation: a theme in search of definition. Monogr Soc Res Child Dev 59(2–3):25–52 7984164

[12] Cicchetti D, Ackerman BP, Izard CE (1995) Emotions and emotion regulation in developmental psychopathology. Dev Psychopathol 7(1):1–10. 10.1017/S0954579400006301

[13] Gratz KL, Roemer L (2004) Multidimensional assessment of emotion regulation and dysregulation: development, factor structure, and initial validation of the difficulties in emotion regulation scale. J Psychopathol Behav Assess 26:41–54. 10.1023/B:JOBA.0000007455.08539.94

[14] Werner K, Gross JJ (2010) Emotion regulation and psychopathology: a conceptual framework. In: Kring AM, Sloan DM (eds) Emotion regulation and psychopathology: a transdiagnostic approach to etiology and treatment. Guilford Press, New York, NY, pp 13–37

[15] Margari F, Craig F, Petruzzelli MG, Lamanna A, Matera E, Margari L (2013) Parents psychopathology of children with attention deficit hyperactivity disorder. Res Dev Disabil 34(3):1036–1043. 10.1016/j.ridd.2012.12.001 23291521

[16] Weiner L, Perroud N, Weibel S (2019) Attention deficit hyperactivity disorder and Borderline personality disorder in adults: a review of their links and risks. Neuropsychiatr Dis Treat 15:3115–3129. 10.2147/NDT.S192871 PMC6850677 31806978

[17] Bunford N, Evans SW, Wymbs F (2015) ADHD and emotion dysregulation among children and adolescents. Clin Child Fam Psychol Rev 18(3):185–217. 10.1007/s10567-015-0187-5 26243645

[18] Chapman AL (2019) Borderline personality disorder and emotion dysregulation. Dev Psychopathol 31(3):1143–1156. 10.1017/S0954579419000658 31169118

[19] Bartsch DR, Roberts RM, Davies M, Proeve M (2015a) The impact of parental diagnosis of borderline personality disorder on offspring: learning from clinical practice. Personality Mental Health 9(1):33–43. 10.1002/pmh.1274 25200499

[20] Barnow S, Spitzer C, Grabe HJ, Kessler C, Freyberger HJ (2006) Individual characteristics, familial experience, and psychopathology in children of mothers with borderline personality disorder. J Am Acad Child Adolesc Psychiatry 45(8):965–972. 10.1097/01.chi.0000222790.41853.b9 16865039

[21] Bartsch DR, Roberts RM, Davies M, Proeve M (2015b) Borderline personality disorder and parenting: clinician perspectives. Adv Mental Health 13(2):113–126. 10.1080/18387357.2015.1065554

[22] Johnston C, Mash EJ, Miller N, Ninowski JE (2012) Parenting in adults with attention-deficit/hyperactivity disorder (ADHD). Clin Psychol Rev 32(4):215–228. 10.1016/j.cpr.2012.01.007 PMC4838457 22459785

[23] Weiss M, Hechtman L, Weiss G (2000) ADHD in parents. J Am Acad Child Adolesc Psychiatry 39(8):1059–1061. 10.1097/00004583-200008000-00023 10939236

[24] Sonuga-Barke EJ, Daley D, Thompson M (2002) Does maternal ADHD reduce the effectiveness of parent training for preschool children’s ADHD? J Am Acad Child Adoles Psychiatry 41(6):696–702. 10.1097/00004583-200206000-00009 12049444

[25] Biederman J, Monuteaux MC, Mick E, Spencer T, Wilens TE, Silva JM, Snyder LE, Faraone SV (2006) Young adult outcome of attention deficit hyperactivity disorder: a controlled 10-year follow-up study. Psychol Med 36(2):167–179. 10.1017/S0033291705006410 16420713

[26] Faraone SV, Doyle AE (2000) Genetic influences on attention deficit hyperactivity disorder. Curr Psychiatry Rep 2(2):143–146. 10.1007/s11920-000-0059-6 11122947

[27] Feldman RB, Zelkowitz P, Weiss M, Vogel J, Heyman M, Paris J (1995) A comparison of the families of mothers with borderline and nonborderline personality disorders. Compr Psychiatr 36(2):157–163. 10.1016/s0010-440x(95)90110-8 7758301

[28] Küng AL, Pham E, Cordera P, Hasler R, Aubry JM, Dayer A, Perroud N, Piguet C (2019) Psychiatric disorders among offspring of patients with bipolar and Borderline Personality Disorder. J Clin Psychol 75(10):1810–1819. 10.1002/jclp.22817 PMC6771911 31268172

[29] Weiss M, Zelkowitz P, Feldman RB, Vogel J, Heyman M, Paris J (1996) Psychopathology in offspring of mothers with borderline personality disorder: a pilot study. Can J Psychiatry 41(5):285–290. 10.1177/070674379604100505 8793148

[30] Braaten EB, Rosén LA (2000) Self-regulation of affect in attention deficit-hyperactivity disorder (ADHD) and non-ADHD boys: differences in empathic responding. J Consult Clin Psychol 68(2):313–321. 10.1037/0022-006X.68.2.313 10780132

[31] Maedgen JW, Carlson CL (2000) Social functioning and emotional regulation in the attention deficit hyperactivity disorder subtypes. J Clin Child Psychol 29(1):30–42. 10.1207/S15374424jccp2901_4 10693030

[32] Shaw P, Stringaris A, Nigg J, Leibenluft E (2014) Emotion dysregulation in attention deficit hyperactivity disorder. Am J Psychiatry 171(3):276–293. 10.1176/appi.ajp.2013.13070966 PMC4282137 24480998

[33] Strine TW, Lesesne CA, Okoro CA, McGuire LC, Chapman DP, Balluz LS, Mokdad AH (2006) Emotional and behavioral difficulties and impairments in everyday functioning among children with a history of attention-deficit/hyperactivity disorder. Prev Chronic Dis 3(2):A52 PMC1563970 16539793

[34] Kaufman EA, Puzia ME, Mead HK, Crowell SE, McEachern A, Beauchaine TP (2017) Children’s emotion regulation difficulties mediate the Association between Maternal Borderline and antisocial symptoms and youth behavior problems over 1 year. J Personal Disord 31(2):170–192. 10.1521/pedi_2016_30_244 PMC11107264 27088167

[35] Suveg C, Shaffer A, Morelen D, Thomassin K (2011) Links between maternal and child psychopathology symptoms: mediation through child emotion regulation and moderation through maternal behavior. Child Psychiatry Hum Dev 42(5):507–520. 10.1007/s10578-011-0223-8 21484417

[36] Cummings EM, Davies PT (1994) Maternal depression and child development. J Child Psychol Psychiatry Allied Discip 35(1):73–112. 10.1111/j.1469-7610.1994.tb01133.x 8163630

[37] Crandall A, Deater-Deckard K, Riley AW (2015) Maternal emotion and cognitive control capacities and parenting: a conceptual framework. Dev Review: DR 36:105–126. 10.1016/j.dr.2015.01.004 PMC4445866 26028796

[38] Morris AS, Silk JS, Steinberg L, Myers SS, Robinson LR (2007) The role of the family context in the development of emotion regulation. Soc Dev 16(2):361–388. 10.1111/j.1467-9507.2007.00389.x PMC2743505 19756175

[39] Blair BL, Perry NB, O’Brien M, Calkins SD, Keane SP, Shanahan L (2014) The indirect effects of maternal emotion socialization on friendship quality in middle childhood. Dev Psychol 50(2):566–576. 10.1037/a0033532 PMC4145591 23795555

[40] Zeman J, Cassano M, Perry-Parrish C, Stegall S (2006) Emotion regulation in children and adolescents. J Dev Behav Pediatrics: JDBP 27(2):155–168. 10.1097/00004703-200604000-00014 16682883

[41] Eisenberg N, Cumberland A, Spinrad TL (1998) Parental socialization of emotion. Psychol Inq 9(4):241–273. 10.1207/s15327965pli0904_1 PMC1513625 16865170

[42] Denham SA, Bassett H, Hamada, Wyatt TM (2010) Gender differences in the socialization of preschoolers’ emotional competence. In A. Kennedy Root & S. Denham (Eds.), The role of gender in the socialization of emotion: Key concepts and critical issues. New Directions for Child and Adolescent Development, 128, 29–49. San Francisco: Jossey-Bass 10.1002/cd.267 20552658

[43] Denham SA, Bassett HH, Wyatt T (2007) The socialization of emotional competence. In: Grusec JE, Hastings PD (eds) Handbook of socialization. Theory and research. The Guilford Press, New York, pp 614–637

[44] Eisenberg N, Fabes RA, Murphy BC (1996) Parents’ reactions to children’s negative emotions: relations to children’s social competence and comforting behavior. Child Dev 67(5):2227–2247 9022240

[45] Denham SA, Workman E, Cole PM, Weissbrod C, Kendziora KT, Zahn-Waxler C (2000) Prediction of externalizing behavior problems from early to middle childhood: the role of parental socialization and emotion expression. Dev Psychopathol 12(1):23–45. 10.1017/s0954579400001024 10774594

[46] O’Neal CR, Magai C (2005) Do parents respond in different ways when children feel different emotions? The emotional context of parenting. Dev Psychopathol 17(2):467–487. 10.1017/s0954579405050224 16761554

[47] Breaux RP, Brown HR, Harvey EA (2017) Mediators and moderators of the relation between parental ADHD symptomatology and the Early Development of Child ADHD and ODD symptoms. J Abnorm Child Psychol 45(3):443–456. 10.1007/s10802-016-0213-1 PMC5357150 27752934

[48] Elgar FJ, Mills RS, McGrath PJ, Waschbusch DA, Brownridge DA (2007) Maternal and paternal depressive symptoms and child maladjustment: the mediating role of parental behavior. J Abnorm Child Psychol 35(6):943–955. 10.1007/s10802-007-9145-0 17577659

[49] van der Bruggen CO, Stams GJ, Bögels SM (2008) Research review: the relation between child and parent anxiety and parental control: a meta-analytic review. J Child Psychol Psychiatry Allied Discip 49(12):1257–1269. 10.1111/j.1469-7610.2008.01898.x 18355216

[50] Katz LF, Windecker-Nelson B (2004) Parental meta-emotion philosophy in families with conduct-problem children: links with peer relations. J Abnorm Child Psychol 32(4):385–398. 10.1023/b:jacp.0000030292.36168.30 15305544

[51] Suveg C, Sood E, Barmish A, Tiwari S, Hudson JL, Kendall PC (2008) I’d rather not talk about it: emotion parenting in families of children with an anxiety disorder. J Family Psychology: JFP: J Div Family Psychol Am Psychol Association (Division 43) 22(6):875–884. 10.1037/a0012861 PMC2610353 19102608

[52] Martel MM (2009) Research review: a new perspective on attention-deficit/hyperactivity disorder: emotion dysregulation and trait models. J Child Psychol Psychiatry Allied Discip 50(9):1042–1051. 10.1111/j.1469-7610.2009.02105.x 19508495

[53] Steinberg EA, Drabick DA (2015) A developmental psychopathology perspective on ADHD and comorbid conditions: the role of emotion regulation. Child Psychiatry Hum Dev 46(6):951–966. 10.1007/s10578-015-0534-2 25662998

[54] Breaux RP, McQuade JD, Harvey EA, Zakarian RJ (2018) Longitudinal associations of parental emotion socialization and children’s emotion regulation: the moderating role of ADHD symptomatology. J Abnorm Child Psychol 46(4):671–683. 10.1007/s10802-017-0327-0 28710531

[55] Gair SL, Brown HR, Breaux R, Lugo-Candelas CI, McDermott JM, Harvey EA (2022) Neural activity and emotion socialization as predictors of later emotion regulation difficulties in children with and without Hyperactivity/Impulsivity. J Atten Disord 26(12):1668–1681. 10.1177/10870547221092171 35510641

[56] Herbert SD, Harvey EA, Roberts JL, Wichowski K, Lugo-Candelas CI (2013) A randomized controlled trial of a parent training and emotion socialization program for families of hyperactive preschool-aged children. Behav Ther 44(2):302–316. 10.1016/j.beth.2012.10.004 23611079

[57] Smit S, Mikami AY, Normand S, Psychopathology (2022) 50(1), 101–115. 10.1007/s10802-021-00818-9 34037888

[58] Havighurst S, Wilson KR, Harley AE, Prior M, Kehoe C (2010) Tuning into kids: improving emotion socialization practices in parents of preschool children- findings from a community trial. J Child Psychol Psychiatry 51:1342–1350. 10.1111/j.1469-7610.2010.02303.x 20735794

[59] Kessler RC, Adler L, Ames M, Demler O, Faraone S, Hiripi E, Howes MJ, Jin R, Secnik K, Spencer T, Ustun TB, Walters EE (2005) The World Health Organization Adult ADHD Self-Report Scale (ASRS): a short screening scale for use in the general population. Psychol Med 35(2):245–256. 10.1017/s0033291704002892 15841682

[60] Doğan S, Öncü B, Küçükgöncü S (2009) Erişkin dikkat eksikliği hiperaktivite bozukluğu kendi bildirim ölçeği (ASRS-v1.1): Türkçe Formunun geçerlilik ve güvenilirliği. Anadolu Psikiyatri Dergisi 10(2):77–87

[61] Leichsenring F (1999) Development and first results of the Borderline personality inventory: a self-report instrument for assessing borderline personality organization. J Pers Assess 73(1):45–63. 10.1207/S15327752JPA730104 10497801

[62] Aydemir Ö, Demet MM, Danacı AE, Deveci A, Taşkın EO, Mızrak S, Şimşek E, İçelli İ (2006) Borderline kişilik envanterinin Türkçe’ye uyarlanması, güvenilirlik ve geçerliliği. Türkiye’de Psikiyatri 8(1):6–10

[63] Turgay A (1994) Disruptive behavior disorders child and adolescent screening and rating scales for children, adolescents, parents and teachers. Integrative Therapy Institute Publication, West Bloomfield (Michigan)

[64] Ercan ES, Amado S, Somer O, Çıkoğlu S (2001) Dikkat eksikliği hiperaktivite bozukluğu ve yıkıcı davranım bozuklukları için bir test bataryası geliştirme çabası. Çocuk ve Gençlik Ruh Sağlığı Dergisi 8(3):132–144

[65] Fabes RA, Eisenberg N, Bernzweig J (1990) Coping with children’s negative emotions Scale (CCNES). Description and scoring. Arizona State University (unpublished manuscript), Tempe, AZ

[66] Yağmurlu B, Altan Ö (2009) Maternal Socialization and child temperament as predictors of emotion regulation in Turkish preschoolers. Infant Child Dev, 275–296

[67] Bjureberg J, Ljótsson B, Tull MT, Hedman E, Sahlin H, Lundh LG, Bjärehed J, DiLillo D, Messman-Moore T, Gumpert CH, Gratz KL (2016) Development and validation of a brief version of the difficulties in emotion regulation scale: the DERS-16. J Psychopathol Behav Assess 38(2):284–296. 10.1007/s10862-015-9514-x PMC4882111 27239096

[68] Yiğit İ, Güzey-Yiğit M (2019) Psychometric properties of Turkish version of difficulties in emotion regulation scale-brief form (DERS-16). Curr Psychol 1–9. 10.1007/s12144-017-9712-7

[69] Garnefski N, Kraaij V (2007) The cognitive emotion regulation questionnaire. Eur J Psychol Assess 23(3):141–149

[70] Akfirat ON, Turan O (2021) Adapting the scale of cognitive emotion regulation strategies for children to Turkish: validity and reliability studies. Int Online J Educ Teach 8(2):904–915

[71] O’brien RM (2007) A caution regarding rules of thumb for variance inflation factors. Qual Quant 41:673–690. 10.1007/s11135-006-9018-6

[72] Hayes AF, Rockwood NJ (2017) Regression-based statistical mediation and moderation analysis in clinical research: observations, recommendations, and implementation. Behav Res Ther 98:39–57. 10.1016/j.brat.2016.11.001 27865431

[73] Tung I, Brammer WA, Li JJ, Lee SS (2015) Parenting Behavior mediates the Intergenerational Association of Parent and child offspring ADHD symptoms. J Clin Child Adolesc Psychology: Official J Soc Clin Child Adolesc Psychol Am Psychol Association Div 53(5):787–799. 10.1080/15374416.2014.913250 PMC4755584 24926775

[74] Kiel EJ, Viana AG, Tull MT, Gratz KL (2017) Emotion socialization strategies of mothers with borderline personality disorder symptoms: the role of maternal emotion regulation and interactions with infant temperament. J Personal Disord 31(3):399–416 10.1521/pedi_2016_30_256 27322579

[75] Bjørk RF, Havighurst SS, Pons F, Karevold EB (2020) Pathways to behavior problems in Norwegian kindergarten children: the role of parent emotion socialization and child emotion understanding. Scand J Psychol 61(6):751–762 10.1111/sjop.12652 32567705

[76] Levine D, Marziali E, Hood J (1997) Emotion processing in borderline personality disorders. J Nerv Ment Dis 185(4):240–246 10.1097/00005053-199704000-00004 9114809

[77] Elliot RL, Campbell L, Hunter M, Cooper G, Melville J, McCabe K, Newman L, Loughland C (2014) When I look into my baby’s eyes. Infant emotion recognition by mothers with borderline personality disorder. Infant Mental Health J 35(1):21–32. 10.1002/imhj.21426 25424403

[78] Meyer B, Pilkonis PA, Beevers CG (2004) What’s in a (neutral) face? Personality disorders, attachment styles, and the appraisal of ambiguous social cues. J Personal Disord 18(4):320–336. 10.1521/pedi.2004.18.4.320 15342321

[79] Murphy D (2006) Theory of mind in Asperger’s syndrome, schizophrenia and personality disordered forensic patients. Cogn Neuropsychiatry 11(2):99–111. 10.1080/13546800444000182 16537236

[80] Baumrind D (1966) Effects of authoritative parental control on child behavior. Child Dev 37(4):887–907. 10.2307/1126611

[81] McGee, R., Wolfe, D., & Olson, J. (2001). Multiple maltreatment, attribution of blame, and adjustment among adolescents. Development and psychopathology, 13(4), 827–846. 11771910

[82] Rabiner DL, Anastopoulos AD, Costello J, Hoyle RH, Swartzwelder HS (2008) Adjustment to college in students with ADHD. J Atten Disord 11(6):689–699. 10.1177/1087054707305106 17712172

[83] Maier SF, Seligman ME (1976) Learned helplessness: theory and evidence. J Exp Psychol Gen 105(1):3

[84] Milich R, Okazaki M (1991) An examination of learned helplessness among attention-deficit hyperactivity disordered boys. J Abnorm Child Psychol 19(5):607–623. 10.1007/BF00925823 1770188

[85] Molina MF, Musich FM (2016) Perception of parenting style by children with ADHD and its relation with inattention, hyperactivity/impulsivity and externalizing symptoms. J Child Fam stud 25:1656–1671

[86] Pearl AM, French BF, Dumas JE, Moreland AD, Prinz R (2014) Bidirectional effects of parenting quality and child externalizing behavior in predominantly single parent, under-resourced African American families. J Child Fam stud 23(2):177–188. 10.1007/s10826-012-9692-z

[87] Smith CL, Calkins SD, Keane SP, Anastopoulos AD, Shelton TL (2004) Predicting stability and change in toddler behavior problems: contributions of maternal behavior and child gender. Dev Psychol 40(1):29–42. 10.1037/0012-1649.40.1.29 14700462

[88] Altan-Aytun Ö, Yagmurlu B, Yavuz HM (2013) Turkish mothers’ coping with children’s negative emotions: a brief report. J Child Fam stud 22:437–443

[89] Fabes RA, Leonard SA, Kupanoff K, Martin CL (2001) Parental coping with children’s negative emotions: relations with children’s emotional and social responding. Child Dev 72:907–920. 10.1111/1467-8624.00323 11405590

